# Historical terminology and superior mesenteric artery syndrome

**DOI:** 10.1016/j.ijscr.2019.12.043

**Published:** 2020-01-23

**Authors:** Steven H. Yale, Halil Tekiner, Eileen S. Yale

**Affiliations:** University of Central Florida College of Medicine, 6850 Lake Nona Blvd, Orlando, FL 32827, USA; Department of the History of Medicine and Ethics, Erciyes University School of Medicine, Talas, Kayseri 38280, Turkey; University of Florida, Division of General Internal Medicine, 2000 SW Archer Rd. Gainesville, FL 32608, USA

To the Editor,

We read with interest the case of Wilkie’s syndrome by Frongia et al. [1]. A variety of terms, many which incorporates the word, obstruction, compression, paralysis, ileus, or incarceration have been used to define this clinical entity. Other terms include megaduodenum, chronic duodenal pseudobstruction or cast syndrome [2]. However, only one, Wilkie’s syndrome, is eponymously named and along with superior mesenteric artery syndrome are the current terminology. Herein, we aim to aim to clarify the historical content and evolution of this disease and attributed terminology.

Beginning from the 1840s, different terms have been used for more than a century to describe the phenomena of proximal intestinal obstruction and dilation caused by compression of the third portion of duodenum by the superior mesenteric artery (SMA) or more specifically between the SMA and aorta. Carl von Rokitansky (1804–1878) initially referenced to this under the name “internal hernias” that lead to incarceration [3]. Nearly 20 years later, he defined this condition as an “internal incarceration” or intestinal incarceration and more thoroughly described it as:

The incarceration is due to the pressure which a portion of the intestine and its mesentery exert on a piece of intestine, bearing on it and compressing it from the front to the rigid posterior abdominal wall. (⋯) Here compression of the lower transverse part of the duodenum is caused by the small intestine mesentery, namely the mesentery artery, which enters the superior mesentery root with the surrounding nerve plexus compressing the S-loop or the last portion of the ileum by a downward displacement of the small intestine [4] (p. 187)

In 1908 in his paper “Acute dilation of the stomach and anterior-mesenteric ileus”, Laffer drew attention to the variety of names that had been in vogue to describe this clinical entity including: acute dilation of the stomach, anterio-mesenteric ileus, gastro-mesenteric ileus, mesenteric intestinal obstruction, post-operative ileus, post-operative arterio-mesenteric intestinal obstruction, post-operative acute dilation of the stomach, acute duodeno-jejunal intestinal obstruction, post-operative gastric paralysis, combined iluesu, duodenal ileus and duodenal compression [5]. Other terms which have been used includes acute gastroduodenal obstruction and mesenteric duodenal compression. It was David Wilkie (1882–1938) while at the University of Edinburgh, who named it chronic duodenal obstruction in 1921 [6]. The eponym Wilkie’s (Wilke’s) syndrome or duodenal ileus arterio-mesenteric ileus was first used by Grauer in 1948 to honor Wilkie’s accomplishment in being the first to provide a comprehensive description of this disease in 75 patients in 1927 [7,8]. Cast syndrome was used by Dorph in 1950 to describe signs and symptoms caused by compression of the abdomen by a hip spic cast or full body cast [9]. Finally, SMA syndrome was used by Kaiser et al. in 1960 [10]. Changes in nomenclature throughout time reflect physicians deeper understanding of the clinical symptoms and pathogenesis of the disease. Of all the terms the latter, superior mesenteric artery syndrome most closely defines this clinical entity accounting for the pathogenesis. Thus, with the exception of Wilkie’s syndrome the remainder terms are synonyms not eponyms, the latter a honorific term ascribed to a person for a discovery.

## Sources of funding

None.

## Ethical approval

Not applicable.

## Consent

Not Applicable.

## Author contribution

Writing and editing manuscript: Steven Yale MD, Halil Tekiner PhD, Eileen S. Yale MD.

## Registration of research studies

Not Applicable.

## Guarantor

Steven Yale MD.

## Declaration of Competing Interest

The authors report not conflicts of interest.

## References

[1] Frongia G., Schenk J.P., Schaible A., Sauer P., Mehrabi A., Günther P. (2019). Food fear, quick satiety and vomiting in a 16 years old girl: it’s bulimia, or maybe not...? A case report of Wilkie’s syndrome (superior mesenteric artery syndrome). Int. J. Surg. Case Rep..

[2] Sturtveant M. (1939). Megaduodenum and duodenal obstruction 1: criteria for diagnosis. Radiology.

[3] Rokitansky C. (1842). Innere-Hernien.

[4] Rokitansky C. (1861). Incarceration.

[5] Laffer W.B. (1908). Acute dilation of the stomach and arterio-mesenteric ileus. Ann. Surg..

[6] Wilkie D.P.D. (1921). Chronic duodenal ileus. Br. Med. J..

[7] Wilkie D.P.D. (1927). Chronic duodenal ileus. Am. J. Med. Sci..

[8] Grauer F.W. (1948). Duodenal ileus (Wilkie’s syndrome) arterio-mesenteric ileus. Bull. Vanc. Med. Assoc..

[9] Dorph M.H. (1950). The cast syndrome: review of the literature and report of a case. N. Engl. J. Med..

[10] Kaiser G.C., McKain J.M., Shumaker J.B. (1960). The superior mesenteric artery syndrome. Surg. Gynecol. Obstet..

